# Maternal antibodies protect offspring from severe influenza infection and do not lead to detectable interference with subsequent offspring immunization

**DOI:** 10.1186/s12985-017-0787-4

**Published:** 2017-06-26

**Authors:** Joan E. M. van der Lubbe, Jessica Vreugdenhil, Sarra Damman, Joost Vaneman, Jaco Klap, Jaap Goudsmit, Katarina Radošević, Ramon Roozendaal

**Affiliations:** 1 Janssen Vaccines and Prevention, Pharmaceutical Companies of Johnson and Johnson, Archimedesweg 4-6, 2333CN Leiden, The Netherlands; 2 Janssen Prevention Center, Center of Excellence of Janssen Research & Development, Pharmaceutical companies of Johnson and Johnson, Leiden, The Netherlands; 3 Present address: Sanofi, Biologics Research, Vitry-sur-Seine, France

**Keywords:** Influenza virus, Seasonal influenza vaccination, Vaccination during pregnancy, Protection, Vaccination gap, Maternal antibodies, Placental transfer, Passive protection

## Abstract

**Background:**

Various studies have shown that infants under the age of 6 months are especially vulnerable for complications due to influenza. Currently there are no vaccines licensed for use in this age group. Vaccination of pregnant women during the last trimester, recommended by the WHO as protective measure for this vulnerable female population, may provide protection of newborns at this early age. Although it has been observed that maternal vaccination can passively transfer protection, maternal antibodies could possibly also interfere with subsequent active vaccination of the offspring.

**Methods:**

Using a mouse model, we evaluated in depth the ability of maternal influenza vaccination to protect offspring and the effect of maternal immunization on the subsequent influenza vaccination of the offspring. By varying the regimen of maternal immunization we explored the impact of different levels of maternal antibodies on the longevity of these antibodies in their progeny. We subsequently assessed to what extent maternal antibodies can mediate direct protection against influenza in their offspring, and whether these antibodies interfere with protection induced by active vaccination of the offspring.

**Results:**

The number of immunizations of pregnant mice correlates to the level and longevity of maternal antibodies in the offspring. When these antibodies are present at time of influenza challenge they protect offspring against lethal influenza challenge, even in the absence of detectable HAI titers. Moreover, no detectable interference of passively-transferred maternal antibodies on the subsequent vaccination of the offspring was observed.

**Conclusion:**

In the absence of a licensed influenza vaccine for young children, vaccination of pregnant women is a promising measure to provide protection of young infants against severe influenza infection.

**Electronic supplementary material:**

The online version of this article (doi:10.1186/s12985-017-0787-4) contains supplementary material, which is available to authorized users.

## Background

Influenza remains a major cause of morbidity and mortality world-wide each year. Previous influenza pandemics including the recent 2009 swine flu pandemic have shown that pregnant women and young children under 6 years of age are at increased risk of complications from influenza infection [1–4]. During pregnancy the immune system of women is modulated to promote fetal tolerance, resulting in an observed excess of influenza-associated deaths in pregnant women [5, 6]. Due to this, the CDC Advisory Committee on Immunization Practices (ACIP) recommended vaccination against influenza for this vulnerable group since 2003, irrespective of the trimester of the pregnancy. In addition, influenza infection during pregnancy has also been implicated in causing various adverse events to the fetus such as congenital malformations, lower birth weight and a significant risk of schizophrenia later in life [7–10]. A number of countries, including the USA [11], therefore additionally recommends to vaccinate all children between 6 months and 5 years of age. In spite of this recommendation, of the children hospitalized with laboratory-confirmed influenza, about half were aged between 0 and 5 months resulting in the highest hospitalization rate among children [1, 12–16]. While vaccination is the main preventative countermeasure against influenza, there are currently no vaccines licensed for use in this vulnerable group of infants <6 months of age. Thus very young infants are at high risk of complications due to influenza in the period between birth and until vaccination becomes possible [17], which is commonly referred to as the vaccination gap.

One possible solution to close the so-called vaccination gap, and to protect infants <6 months of age, is by passive protection via maternal antibodies. Maternal antibodies are transferred from the mother to the child mostly via the placenta during pregnancy (IgG antibody subtype) and to a lesser extent via breast milk (mostly IgA). Increasing the level of influenza-specific maternal antibodies by vaccination of females during pregnancy has been shown to confer protection to their progeny in various animal models such as ferrets [18], pigs [19] and mice [20, 21]. Although in mice it has been observed that maternal vaccination transferred protection against lethal influenza challenge, the protection transferred did not appear to be related to the level nor longevity of maternal antibodies [22–25]. Additionally, several studies show that maternal antibodies may have a negative impact on active immunization [19, 26–28]. It is generally thought that maternal antibodies binding to the vaccine antigen are masking the epitopes from the B cells of the child, thereby dampening its immune response.

Finally, while studies in humans suggest the potential of maternal vaccination to decrease influenza illness in newborns [29, 30], there is a large heterogeneity in outcome among studies. This difference in outcomes is likely due to the fact that morbidity is frequently assessed on clinical symptoms such as Influenza-like-illness (ILI) rather than laboratory confirmed influenza. Poehling et al. recently showed in a study including over 1500 infants hospitalized with respiratory symptoms and fever that laboratory-confirmed influenza could only be detected in 10% of the children [1].

We decided to further characterize the impact of maternal influenza vaccination on the offspring in a mouse model. By varying the vaccination regimen given to pregnant females we explore the impact of different levels of maternal antibodies induced on the longevity of these antibodies in their progeny. We then assess to what extent maternal antibodies can transfer protection against lethal influenza exposure in their offspring. Subsequently we study whether maternal antibodies interfere with protection induced by active vaccination of their offspring.

## Methods

### Immunization and mating of mice

Thirteen-week-old female mice (BALB/c, Charles River) were subcutaneously (s.c.) immunized in the scruff of the neck with 100 μl of seasonal trivalent vaccine (Inflexal) containing 3 μg HA per influenza strain, at an interval of 3 weeks when receiving multiple immunizations. One day before each immunization, a pre-immunization blood sample was obtained via submandibular bleeding to assess immunization efficacy. One week prior to the last immunization the female mice were mated with unimmunized male BALB/c mice, aged 17 to 37 weeks, two females to one male in the same cage. Females were separated from the males when a mating plug was observed, or after 4 nights. Three weeks after mating most female mice gave birth and pups of both sexes were randomly distributed within treatment groups. Infant mice were subcutaneously immunized in the scruff of the neck with 50 μl PBS or seasonal trivalent vaccine (Inflexal), diluted in PBS when applicable. Infant mice were weaned and separated from their mothers at the age of 3 weeks before immunization.

### Influenza challenge

At the age of 7 weeks offspring was challenged an H1N1 influenza strain. A stock of H1N1 A/Netherlands/602/2009 (Viroclinics) was grown on embryonated chicken eggs. Anaesthetized mice were challenged intranasally with 25xLD50 virus in a total volume of 50 μl, 25 μl per nostril. Groups of mice (*n* = 20 and *n* = 8) receiving either PBS or a broadly protective monoclonal antibody (CR6261, 15 mg/kg in PBS) intramuscularly were used as negative and positive controls for challenge models, respectively. Challenges were considered valid when there was a statistically significant difference in survival proportion (Fisher’s exact-test, 2-sided) between the control groups (data not shown). Bodyweight and clinical score were monitored daily for up to 21 days or until a humane endpoint to limit animal discomfort.

Humane endpoint was defined based on clinical score as is established practice [31–33]. Our experience with Influenza challenge models suggests that using alternative humane endpoints, such as bodyweight-loss would lead to underestimation of protection. The amount of bodyweight-loss before animals reach clinical score 4 is variable. We explicitly discussed this issue with our Ethical Review Board, and they agreed to allow clinical score 4 as a humane endpoint. Clinical scores were defined as: 0 = no clinical signs, 1 = rough coat, 2 = rough coat, less reactive, passive during handling, 3 = rough coat, rolled up, labored breathing, passive during handling, 4 = humane endpoint: rough coat, rolled up, labored breathing and unresponsive. All mouse studies were approved by DEC Consult (Independent ethical institutional review board).

Repeated measurements in the challenge phase (bodyweight and clinical scores) were summarized as a single outcome per animal using an area under the curve (AUC) approach where missing values for animals that died early were imputed with a last-observation-carried-forward method. See Additional file 1: Figures. S3, S4 and S5 for changes in bodyweight and observed clinical scores.

### recH1 a/California/07/2009 ELISA

Multisorp (Nunc) 96-well flat bottom plates were coated overnight with rH1 A/California/07/2009 (Protein Science Inc., 0.5 μg/ml), washed and blocked. Wells were incubated with duplicate serial 2-fold dilutions with a start dilution of 1:50 of mouse serum in block buffer (2% skimmed milk in PBS) for 1 h at room temperature, and washed. Wells were incubated with anti-human IgG for the detection of the human monoclonal CR6261 in the positive control group(Jackson ImmunoResearch), or anti-mouse IgG (KPL) conjugated to HRP for 1 h at room temperature, washed and developed using OPD substrate (Thermo Fischer Scientific). OD was read at 492 nm using a Powerwave Synergy plate reader (Biotec), and compared to the standard curve of CR9114 (produced in house), for calculation of ELISA units using a slope based weighted average approach: The OD of each sample dilution was quantified against the standard curve of CR9114 included on each plate. The final concentration per sample was calculated by a weighted average, using the squared slope of the standard curve at the location of each quantification as weight. Negative samples were set at the limit of detection (LOD), defined as the lowest sample dilution multiplied by the lowest standard concentration, with an OD response above the lower asymptote of the standard curve and background. ELISA titers were expressed as log10 ELISA Units (EU) per ml, and are not intended to infer amounts in micrograms. CR9114 is a monoclonal antibody binding to a conserved epitope found on the stem region of group 1 and 2 influenza A viruses and influenza B viruses [31].

### Pseudoparticle neutralization assay

The PhenoSense® neutralization assay using pseudoparticles was conducted at Monogram Biosciences, Inc. The assay uses a single infection round of HEK293cells with replication deficient retroviral vector encoding a firefly luciferase indicator gene and HA and NA A/California/07/09 expression vectors. A fourth expression vector containing a human airway serine protease used in processing the HA protein, TMPRSS2, is also transfected. The resultant pseudoviruses are harvested from culture supernatants, filtered, titered and stored at -80 °C. The pseudovirus stocks, at a concentration giving approximately 30,000–300,000 relative light units (RLU) per well, were incubated at 37 °C for one hour with 3 fold serial dilutions of test antibody (i.e. serum or control mAbs) in a 96 well plate starting at a 1:50 dilution. HEK293 cells are then added to each well and incubated at 37 °C in 5% CO2 for 3 days. Luciferase substrate and cell lysing reagents are added to the plates which are read on a luminometer. Data were provided as half-maximal inhibitory concentration (IC50) values by Monogram Biosciences and are reported as such: Inhibition curves are defined by a four-parameter sigmoidal function and are fit to the data by nonlinear least-squares and bootstrapping. The ability of antibody in the serum to neutralize Influenza infectivity is assessed by measuring luciferase activity in the culture 72 h after viral inoculation as compared to a control infection using a murine leukemia virus envelope (aMLV) pseudotyped virus. Neutralization titers are expressed as the reciprocal of the serum dilution that inhibited the virus infection by 50%. A reaction is called positive for neutralization when there is at least 50% inhibition of infection of an Influenza strain and when there is an IC50 at least 3 fold higher than the IC50, if any, of the same sample tested with the specificity control, aMLV env.

### Hemagglutination inhibition assay

Nonspecific hemagglutination inhibitors were removed from sera by O/N incubation with Cholera Toxin (Sigma-Aldrich; St. Louis, MO, USA; diluted 1:25 in PBS) at 37 °C in presence of turkey red blood cells (bioTRADING Benelux B.V., Mijdrecht, the Netherlands, 0.5% in PBS). Cholera toxin was subsequently inactivated by heat at 56 °C for 30 min. Sera were tested in duplicate 2-fold serial dilutions with an initial dilution of 1:8. Diluted sera were mixed with 8 HA units of H1N1 A/California/07/09 (reassortant NYMC X-181) for 1 h at RT. Turkey red blood cells (1% in PBS) were added to the serum and incubated for 1 h at room temperature (RT). Confirmed positive and negative sera were used as assay controls. HAI titer is expressed as reciprocal of the highest dilution that completely inhibited hemagglutination.

### Statistical methods

In the challenge studies, differences in survival have been analyzed by exact logistic regression while accounting for the correlation of pups from the same nest. Differences in survival duration have been tested as a rank test by Cochran-Mantel-Hänszel test with the nesting included as covariable. The clinical scores have been analyzed as cumulative logistics models f score classes with nest as random factor. The body weight has been analyzed as mixed model of the AUC of the difference in weight compared to baseline with treatment as fixed factor and nest as random factor.

**Fig. 1:**
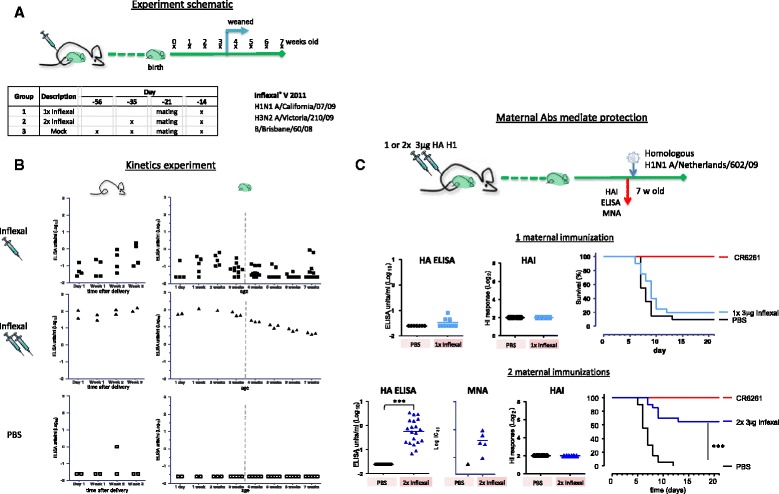
Maternal antibodies mediate protection against lethal influenza challenge. **a** Design; Adult female mice were vaccinated either 1× or 2× with Inflexal, or 3× with PBS before and/or after mating. Blood samples of offspring were collected weekly from the age of 1 day. Number of pups varies due to available litter size. **b** Level and duration of detectable maternal antibodies; ELISA titers against homologous H1 A/California/07/09 in dams and offspring. Pups were weaned at age 3 weeks. Maternal antibodies are present longer and at higher titers when mothers received a prime / boost vaccination with Inflexal. **c** Comparison of protection mediated by maternal antibodies; Adult female mice were vaccinated either 1× or 2× with Inflexal or PBS before and/or during mating. Offspring was challenged with H1N1 A/Netherlands/602/09 at the age of 7 weeks. As challenge controls mice received either PBS or broadly protective monoclonal antibody CR6261 intramuscularly 1 day prior to challenge. One day prior to challenge, at age 7 weeks, blood samples were taken and analyzed for recHA A/California/07/09 binding antibodies in an IgG ELISA assay and H1N1 A/California/07/09 HAI titers. Samples from pups of the mothers which were immunized twice were also tested for H1N1 A/California/07/09 MNA titers in a pseudoparticle assay. An increase in survival was observed when protective maternal antibodies were present in the offspring before challenge as detected by homologous HA ELISA and confirmed by MNA, but in absence of HAI titers. *** *p* < 0.001

In all challenge studies, first a gatekeeper test was performed: the group of which both mother and pups received mock immunization compared the positive challenge control group which received monoclonal anti-influenza antibody CR6261. The studies in Figs. 1c and 3b a step-wise test approach was used: the contrast between the group of which both mothers and pups received TIV immunization compared to both receiving a mock immunization was the primary contrast (without adjustment for multiple testing). If significant all remaining contrasts between groups were tested simultaneously with Bonferroni adjustment for testing 5 contrasts. In the studies in Figs. 3a and 4b (after the gatekeeper) a step-wise adjustment of *p* values was used going from the highest to the lowest dose (3A) or from the most to the least injections (4B).

**Fig. 2:**
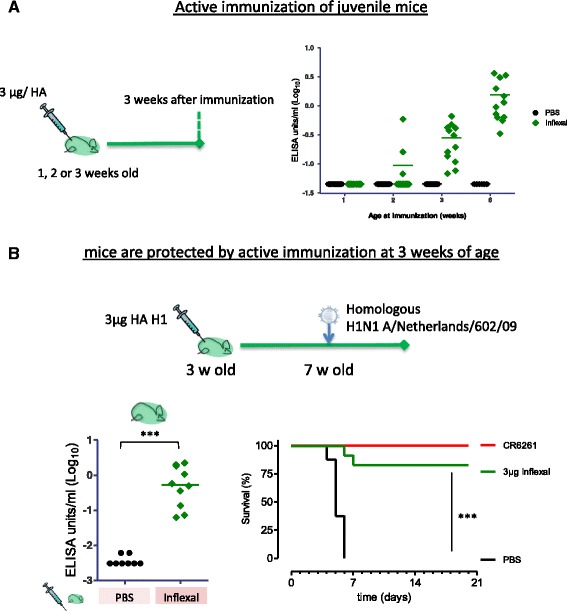
Active immunization protect juvenile mice against lethal influenza challenge. **a** Mice produce detectable antibody responses when immunized at 3 weeks of age; Pups were immunized at either 1, 2 or 3 weeks of age, or 6 weeks as adult control, and blood samples were tested for homologous H1 HA A/California/07/09 antibodies 3 weeks later in an IgG ELISA assay. **b** Immunization of 3 week old pups with 3 μg per HA in the trivalent Inflexal vaccine. Pups were immunized at 3 weeks of age and subsequently challenged with H1N1 A/Netherlands/602/09, a strain homologous to the H1 strain included in the vaccine. Mice were daily weighed and observed for clinical symptoms for 3 weeks. One day prior to challenge, at age 7 weeks, blood samples were taken and analyzed for recHA A/California/07/09 binding antibodies in an IgG ELISA assay (left graph). ***p < 0.001

**Fig. 3:**
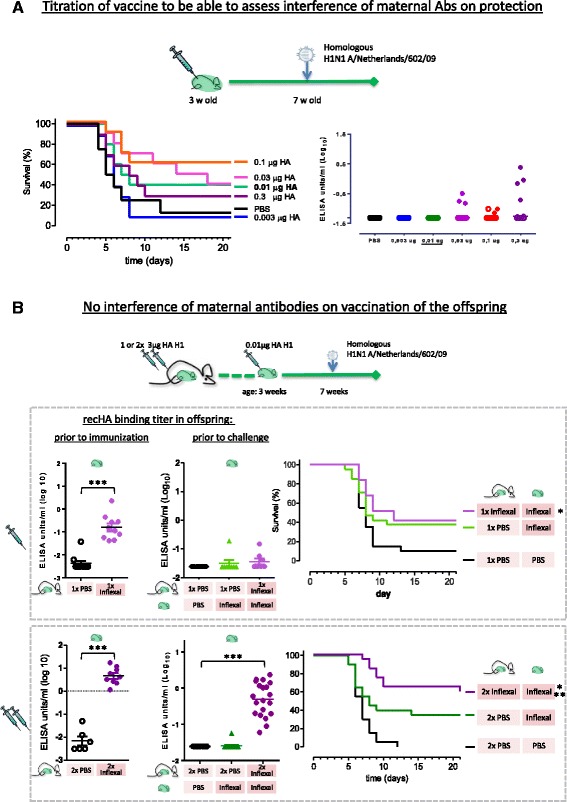
Maternal antibodies do not interfere with vaccine induced protection. **a** At the age of 3 weeks juvenile mice were immunized with trivalent influenza vaccine Inflexal titrated from 0.3 μg HA per strain to 0.003 μg / HA and 4 weeks later challenge with homologous H1N1 A/Netherlands/602/09. Vaccination 0.01 μg H1 HA protects 40% of mice against lethal homologous H1N1 A/Netherlands/602/09 influenza virus challenge. Binding antibody titers 1 day prior to challenge at 7 weeks of age, as measured by recHA A/California/07/09 ELISA assay. Open symbols represents values at LOD. **b** Adult female mice were vaccinated either 1× or 2× with Inflexal or mock immunized with PBS before and/or during pregnancy. Offspring was either vaccinated with Inflexal or PBS at the age of 3 weeks and subsequently challenged with H1N1 A/Netherlands/602/09 at the age of 7 weeks. An increase in survival was observed only when protective maternal antibodies were present just prior to challenge as detected by homologous recHA A/California/07/09 ELISA, see bottom graphs. *p < 0.05, *** *p* < 0.001 statistical significance compared to mock immunization (survival %)

The immunogenicity results were tested by t-test (2 groups) or anova (multiple groups) in case of normal distributed results or by (a set of) Mann-Whitney Rank test(s) if a considerable proportion of the results was at LOD. *P* values from the study in Fig. 2b have been Dunnett adjusted for multiple testing for comparison of set of test groups with one reference group. *P* values from the studies in Figs. 1c and 3b have been Tukey adjusted (anova) or Šidak adjusted (Mann-Whitney) for testing all pair-wise comparisons.

## Results

### The level and longevity of maternal antibodies in offspring correlate to antibody titers in pregnant mice

Adult female mice received one or two injections of seasonal trivalent influenza vaccine Inflexal which included influenza strains A/California/07/2009 (H1N1), A/Victoria/210/2009 (H3N2) and B/Brisbane/60/2008, or were mock immunized with PBS. For each of these regimens, the final immunization was given after mating, with some of the females presumed pregnant (see Fig. 1a for schematic representation). Serum samples were taken weekly from both pregnant and non-pregnant females, and pups from 1 day after delivery. At the age of 3 weeks pups were weaned and separated from their mothers (see design in Fig. 1a). The serum samples were analyzed for the presence of vaccine homologous H1 Hemagglutinin (HA) antibodies in an ELISA assay (see Additional file 1: Figure S1 for the longevity of antibodies in mothers).

In agreement with previous findings in humans [34], immune responses in pregnant females were lower than in non-pregnant female mice (*p* = 0.016, see Additional file 1: Figure S2). Prime/boost immunization trends towards higher titers in dams than prime only (see Additional file 1: Figure S1). This trend of influenza-specific maternal antibodies can also been seen in the pups, when dams received multiple immunizations as compared to a single immunization (Fig. 1b, no formal statistical analysis was performed on these data). The longevity of detectable maternal antibodies was correlated to level of maternal antibodies transferred, i.e. when dams received a single immunization and maternal antibodies levels were relatively low, the antibodies became undetectable within 5 weeks. In contrast, when dams received a prime-boost vaccination regimen, the maternal antibodies were still readily detectable 7 weeks after birth.

Pups were weaned at 3 weeks of age, around the time at which maternal antibodies started to decline, see Additional file 1: Figure S1, suggesting that maternal antibodies were transferred both via the milk and the placenta, as previously described [35].

To study whether maternal antibodies are able to protect offspring against influenza we challenged offspring at 7 weeks of age with a H1N1 A/Netherlands/602/09 (closely related to the H1N1 component of the vaccine). In agreement with the previous experiment, at the age of 7 weeks maternal antibodies had been cleared when dams received a single vaccination, but were detectable in all pups when dams had received a prime-boost vaccination regimen (*p* < 0.001 at age 7 weeks, see Fig. 1c). In addition, we tested whether the antibodies detected in the latter group would be able to neutralize virus. Remaining serum samples of five pups were tested. We found detectable neutralization titers in all these pups, see Fig. 1c. As shown in Fig. 1c, a significant proportion of mice survived challenge (65%, *p* < 0.001) only when mothers received two vaccinations and maternal antibodies were detectable by ELISA assay at time of challenge (*p* < 0.001, see Additional file 1: Figure S3 more information on change in bodyweight and clinical scoring). HAI titers were not detectable at any time prior to challenge.

### Active immunization protects juvenile mice against lethal influenza challenge

Mice before the age of 6 weeks are considered immature and their immune system is not fully developed [36]. To determine from which age immunization induces a detectable IgG antibody response we vaccinated mice at the age of 1, 2 or 3 weeks, using 6 week old mice as a control with a competent immune system. Blood samples were taken 3 weeks later and analyzed for the presence of homologous influenza-specific IgG levels. We found that immunization of mice induced detectable homologous IgG titers in all pups from the age of 3 weeks, see Fig. 2a. In contrast, only 3 of the 14 pups aged 2 weeks had detectable antibody responses 3 weeks after vaccination, and none when immunized at 1 week, suggesting immune system immaturity at this age. Influenza-specific IgG titers observed in pups vaccinated at 3 weeks old were still lower than those observed in adult mice, aged 6 weeks, implying that the immune response at 3 weeks is not fully developed, or has slower kinetics.

To determine whether the antibodies induced by vaccination at 3 weeks of age can induce protection against lethal influenza challenge we inoculated the pups 4 weeks after vaccination with a lethal dose of H1N1 A/California/07/09. We observed 83% survival of pups vaccinated at the age of 3 weeks, see Fig. 2b. We found the level of homologous H1 HA ELISA antibody titers in these pups to be similar to that of pups which received maternal antibodies from dams immunized twice, see Fig. 1c. Moreover, while in actively immunized pups protection may be mediated by both cellular and humoral immunity, the level of protection we observed is also comparable to the pups from prime-boost immunized mothers (see Fig. 1c).

### Maternal immunity does not lead to detectable interference with immunization of offspring

We subsequently investigated whether a level of maternal antibodies associated with direct protection would interfere with vaccine induced protection in young mice. To be able to observe whether maternal antibodies have a positive or negative impact on survival after vaccination, we first determined which dose of vaccine given to the pups elicits partial protection to influenza challenge. We vaccinated juvenile mice at the age of 3 weeks with a sub-optimal dose of vaccine and challenged 4 weeks later with a lethal dose of H1N1 A/Netherlands/602/09 influenza virus. We titrated the dose of trivalent vaccine Inflexal from 0.3 μg per HA to 0.003 μg per HA given to 3 week old pups. Overall protection decreases with a decreasing dose of vaccine, however, there is some overlap between dosages, suggesting a rather flat dose response curve (Fig. 3a). A dose of 0.01 μg/HA, which induced protection in 40% of the pups in absence of detectable HA ELISA titers, was selected for subsequent studies.

A vaccine dose of 0.01 μg/HA was given to pups at 3 weeks of age in absence or presence of maternal antibodies induced by either one or two immunizations, and subsequently challenged with influenza virus 4 weeks later, see Fig. 3b. In agreement with the previous experiment, see Fig. 3a, this dose of vaccine induced survival in approximately 40% of the pups in absence of detectable HA ELISA titers (*p* = 0.031).

No difference in protection of the offspring was observed when the mothers were vaccinated once during pregnancy, and maternal antibodies were present at time of vaccination of the offspring but had waned at time of challenge (42% survival in this group, see Fig. 3b).

On the other hand, two immunizations of pregnant females in combination with vaccination of offspring resulted in significant HA ELISA titers at time of influenza challenge (*p* < 0.001) and induced protection (as defined by survival) in 65% of the offspring (*p* < 0.001). This survival proportion is comparable to that seen without vaccination of the offspring (60%, see Fig. 1c), suggesting that vaccination of the offspring with a low dose of vaccine does not impact the protection transferred by maternal antibodies.

These findings confirm the ability of maternal antibodies to confer protection against lethal influenza challenge. No additional protection was gained by a suboptimal dose of vaccine given to the offspring when maternal antibodies were present at time of challenge. While these results do not formally exclude immuno-interference by maternal antibodies, they show that maternal antibodies can confer protection, even when combined with juvenile vaccination.

### Three maternal immunizations induce a high level of maternal antibodies in the offspring and confer protection against influenza to all offspring

**Fig. 4:**
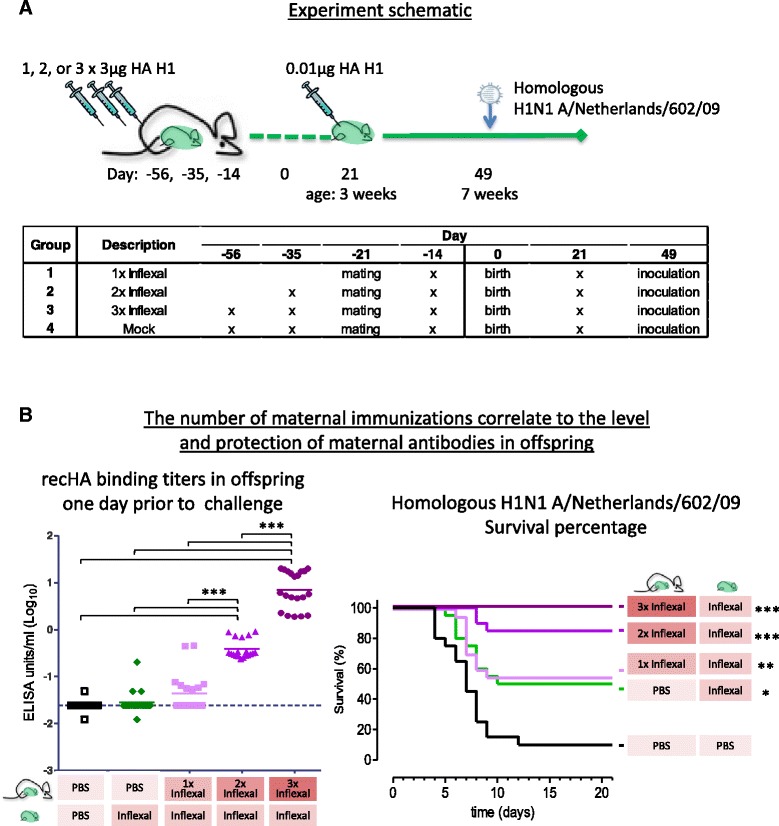
The number of maternal immunizations correlate to the level of maternal antibodies and protection against lethal influenza challenge of the offspring. **a** Design; Adult female mice were vaccinated either 1×, 2× or 3× with Inflexal, or 3× mock immunized with PBS before and/or during mating. Offspring were vaccinated at 3 weeks of age with 0.01 μg Inflexal and subsequently challenged with H1N1 A/Netherlands/602/09 4 weeks post immunization. **b** The level and protection mediated by maternal antibodies correlate to the number of maternal immunizations; Adult female mice were vaccinated either 1×, 2× or 3× with Inflexal or PBS before and/or during mating. Offspring was immunized with 0.01 μg / HA and subsequently challenged with H1N1 A/Netherlands/602/09 at the age of 7 weeks. One day prior to challenge blood samples were analyzed for homologous recHA A/California/07/09 IgG binding titers. Statistical significance of recHA binding titers and survival percentage compared to the mock immunized group, *p < 0.05, *p < 0.01, ***p < 0.001

Results described up to this point indicate that maternal antibodies can confer protection against lethal influenza challenge irrespective of vaccination of the offspring with a suboptimal dose of vaccine (Fig. 3). To test whether the survival of offspring by maternal antibodies can be further increased, we repeated the experiment above and included a group which received three immunizations. As seen in Fig. 4, mothers were given either 1, 2 or 3 vaccinations of which the final immunization was given 1 week after mating. Pups were vaccinated at the age of 3 weeks with a suboptimal dose of vaccine, and challenged 4 weeks later with a lethal dose of H1N1 A/Netherlands/602/09.

In agreement with the previous experiment in Fig. 3b, vaccination of mothers during pregnancy transferred maternal antibodies to the offspring and are present during vaccination of the offspring at the age of 3 weeks, see Fig. 4b. And again, in agreement with the previous experiment, these HA binding maternal antibodies had waned in the offspring at the age of 7 weeks when the mothers had received a single vaccination but were still detectable in all the offspring when mothers received two vaccinations. When offspring was subsequently challenged with a lethal dose of influenza virus at the age of 7 weeks, the level of survival of pups from dams that were immunized twice trended to be higher than that of pups from dams that were immunized only once (85 and 55% resp.). Also, the protection of offspring observed in this experiment is comparable to that observed in the previous experiment (see Fig. 3b: 65% survival in offspring from mothers vaccinated twice and 42% survival in offspring from mothers which received one vaccination). In addition to the previous experiment we included a group of which the mothers received three vaccinations during pregnancy. Interestingly, three immunizations of the mothers, in combination with immunization of the pups at 3 weeks of age with a suboptimal dose of vaccine, further increased the level of homologous HA-binding antibodies in the pups. Even more interesting was the observation that three vaccinations of mothers during pregnancy was able to confer protection against lethal influenza virus challenge to all offspring compared to mock immunization (*p* < 0.001), see Fig. 4b. These results indicate that multiple immunizations of mothers prior to delivery correlate to the level of homologous antibodies found in the offspring and to passive protection transferred against lethal influenza challenge.

## Discussion

There are currently no vaccines licensed for use in infants under 6 months of age. One strategy to protect these very young and to close the gap until vaccination, is by passive protection via maternal immunity.

Our results show that vaccination of pregnant female mice protects their offspring against lethal influenza challenge. The longevity of maternal antibodies in the offspring was correlated to the level of antibodies in the pups at the time of weaning (i.e. at the age of 3 weeks), suggesting that additional boost vaccination of pregnant females could result in longer duration of protection. Moreover we have shown that the presence of maternal antibodies at the moment of vaccination of newborn mice does not appear to inhibit the level of vaccine-induced protection in the offspring when challenged later in life. In agreement with a study of Schlaudecker et al. we observed a dampened immune response to the trivalent seasonal influenza vaccine in pregnant females 3 weeks after delivery relative to females which did not get pregnant [37]. The reason behind this dampened response is likely a result of the immunological changes that occur during pregnancy to prevent rejection of the fetus but might also be explained by behavioral factors such as maternal weight and stress exposure [38]. In spite of the fact that the immune response to the vaccine is dampened in pregnant females, prime/boost vaccination of females during pregnancy resulted in significant protection of offspring against lethal challenge.

Surprisingly, protection by maternal antibodies could be achieved in the absence of detectable hemagglutinin inhibition assay (HAI) titers in the offspring. Influenza viruses are able to agglutinate mammalian or avian red blood cells by binding to cell-surface sialic acids. When influenza HA specific antibodies are present, agglutination is inhibited. The HAI assay is globally used to determine the immunogenicity of vaccines, since it is an accepted correlate of protection. We here show, as suggested before by Reuman et al. [39], that the HAI assay lacks the sensitivity needed to study the protective efficacy of maternal vaccination. In contrast, neutralization titers could be detected 7 weeks after birth in pups of which mothers received two immunizations and these pups were subsequently protected from lethal influenza challenge. In addition to neutralizing antibodies not detected by the HAI assay due to a lack in sensitivity, non-neutralizing antibodies could potentially also play a role in mediating protection via antibody effector mechanisms such as ADCC [40]. Our results show that vaccination of dams confers protection to offspring against lethal influenza challenge when maternal antibodies were detectable by the sensitive H1 IgG HA ELISA assay. In addition, no HA-specific IgA binding antibodies were detected in serum of the offspring 1 day after birth, nor in the serum of their dams after three immunizations with a seasonal vaccine (data not shown). We hypothesize that because the dams were immunized intramuscularly the immune response will be mainly dominated by IgG antibodies. In contrast, influenza vaccines given orally will induce higher levels of mucosal immunity and IgA. In view of the absence of detectable IgA in our system, anti-influenza IgA is unlikely to explain the observed effects.

One strategy to close the current vaccination gap in very young children in which vaccination is not yet possible, is by passive protection via maternal immunity. Maternal vaccination has been shown to transfer passive protection to various infectious diseases, both viral diseases such as measles, as well as bacterial infectious diseases such as pertussis and tetanus [41–43]. Several studies also indicate a decrease in number of influenza related illnesses or a delay of disease onset in offspring in humans when mothers were vaccinated [25, 44–48]. However the effectiveness of vaccination to protect offspring in these studies ranged widely from no protection observed to over 90% due to the lack of an uniform outcome [49]. Especially the use of clinical outcomes, such as physician visits and hospitalization due to acute respiratory infection, or the use of the HAI assay to detect maternal antibodies, led to a variety of estimates of vaccine effectiveness [46–48]. Poehling et al. recently showed in a study including over 1500 infants hospitalized with respiratory symptoms and fever that laboratory-confirmed influenza could only be detected in 10% of the children as shown by the more sensitive rtPCR and/or viral culture [1]. This demonstrates the possible inaccurate vaccine efficacy estimates by studies lacking confirmation of influenza virus infection. Epidemiological studies using more sensitive, influenza-specific assays to confirm both vaccination status of pregnant women (e.g. ELISA), as well as influenza infection of their offspring (e.g. rapid influenza diagnostic test (RIDT) or molecular assays including reverse transcription-polymerase chain reaction (RT-PCR)) will have to be performed to determine the effectiveness of vaccinating mothers during pregnancy.

When vaccination of pregnant women is considered however, a reason of concern to vaccinate is the possible inhibiting effect of maternal antibodies on the immune response of young children to vaccination. Numerous reports show an inhibitory effect of maternal antibodies on the humoral immune response of the offspring after immunization [50]. Siegrist reviewed studies which analyzed this dampening effect in more detail and summarized several potential mechanisms [51]. One hypothesis is that binding of maternal antibodies to the antigens mask the epitopes, thus preventing the priming of the infant B cells. In addition, binding of maternal antibodies to the epitopes of interest may result in rapid clearing of the through Fc-dependent phagocytosis further dampening the immune response of very young children. However, even though dampening of the immune response after primary vaccination has often been observed, immunization in the presence of maternal antibodies does not interfere with a balanced humoral and cellular booster vaccination indicating no effect of maternal antibodies on the formation of a memory response [41]. For example, the immune response in Rhesus macaques was weakened when maternal antibodies were present during vaccination, however, effect on protection from subsequent measles challenge was observed [42]. This observation is in line with our study where pups induced protective immune responses against Influenza to the same degree in the absence as presence of maternal antibodies when induced by one vaccination of the dams during pregnancy. Moreover, we have demonstrated that vaccination of offspring will not contribute to, nor interfere with, protection mediated by maternal antibodies when still present at high levels during influenza exposure.

Whether active influenza vaccination of young children under the age of 6 months is a possible second strategy to protect this group has to be determined. Currently no vaccine is licensed for use at this vulnerable age. Here we have shown that indeed only part of the mice under the age of 3 weeks, which translates to children approximately under the age of 2 months [36], produced a detectable immune response to vaccination. However, albeit resulting in lower levels, all mice at the age of 3 weeks were able to induce homologous IgG ELISA titers to vaccination suggesting vaccination might be possible before full immune system maturity is reached. Moreover, the protective efficacy of these actively induced antibodies did not appear to be influenced by the presence of maternal antibodies at time of vaccination.

## Conclusions

Together our data from a mouse model suggest maternal vaccination as an effective way of protecting offspring early in life. Maternal antibodies correlated to protection in absence of detectable HAI titers and interestingly did not lead to detectable interference with the protective efficacy of vaccination of juvenile mice. Whether our results are comparable to the situation in humans has to be studied in further detail. Sensitive laboratory techniques will have to be used to determine the effectiveness of vaccinating mothers during pregnancy, in view of the fact that HAI titers may not be the only correlate of protection. In addition, we highly recommend clinical studies using laboratory confirmed influenza infection, to avoid inaccurate efficacy estimates. We conclude that passive protection via maternal immunity should be considered as a possible strategy to protect very young infants from influenza and could be combined with early vaccination, when influenza vaccines for young infants become available.

## Additional file

Additional file 1: Supplementary figures. (PPTX 314 kb)

## Abbreviations

ACIP: Advisory Committee on Immunization Practices
CDC: Center for Disease Control
ELISA: Enzyme-linked Immunosorbent Assay
HA: Hemagglutinin
HAI: Hemagglutinin inhibition assay
IgA: Immunoglobulin A
IgG: Immunoglobulin G
RIDT: rapid influenza diagnostic test
RT-PCR: reverse transcription-polymerase chain reaction
WHO: World Health Organization
