# Supplementary material for: Maternal antibodies protect offspring from severe influenza infection and do not lead to detectable interference with subsequent offspring immunization
Source: Virol J. 2017 Jun 26;14:123. doi: 10.1186/s12985-017-0787-4 (PMC5485558; doi:10.1186/s12985-017-0787-4)
Supplement: Additional file 1: — Supplementary figures. (PPTX 314 kb) [file 12985_2017_787_MOESM1_ESM.pptx]

## Slide 1
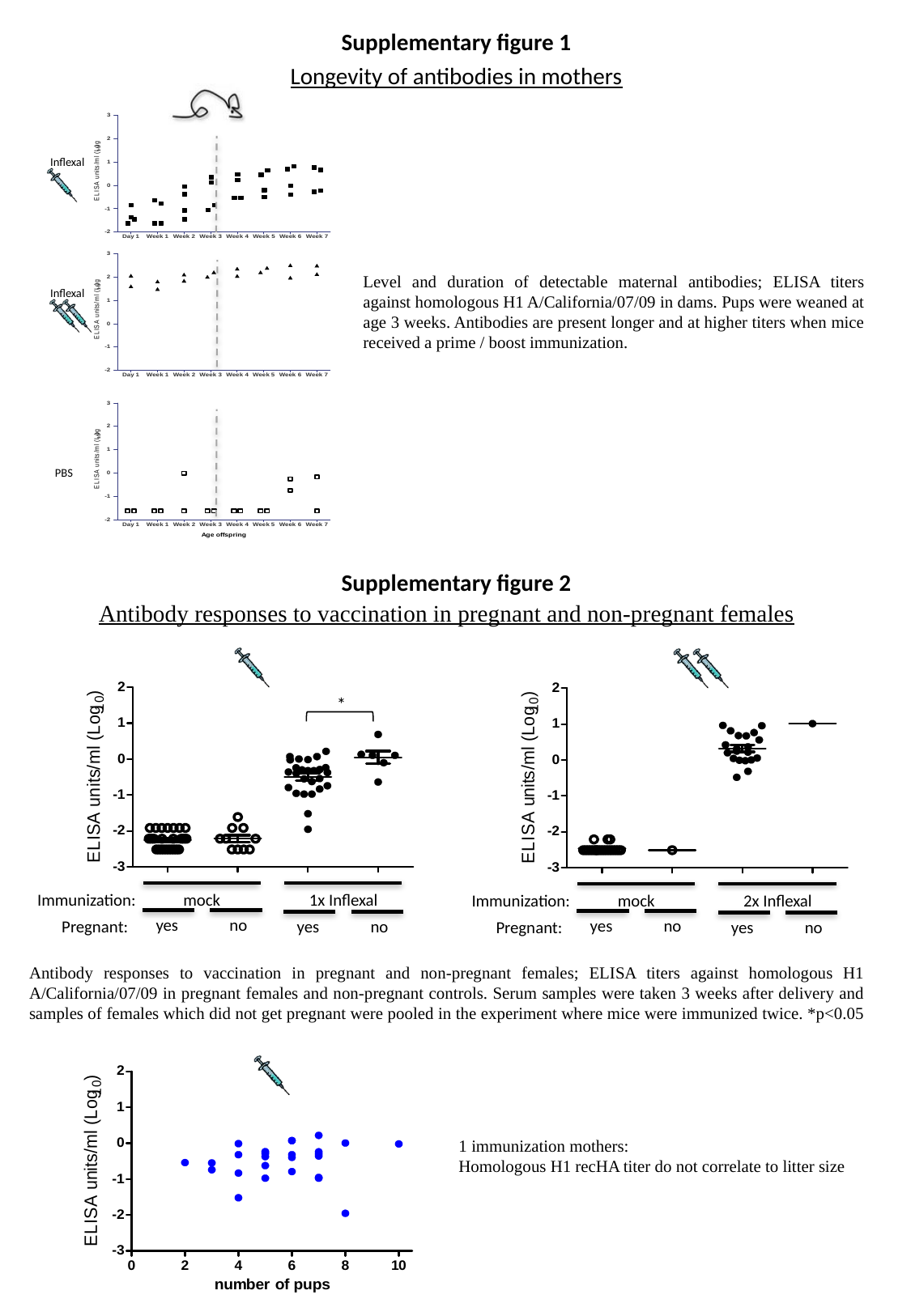

Supplementary figure 1
Longevity of antibodies in mothers
Inflexal
Level and duration of detectable maternal antibodies; ELISA titers against homologous H1 A/California/07/09 in dams. Pups were weaned at age 3 weeks. Antibodies are present longer and at higher titers when mice received a prime / boost immunization.
Inflexal
PBS
Supplementary figure 2
Antibody responses to vaccination in pregnant and non-pregnant females
*
1x Inflexal
Immunization:
mock
2x Inflexal
Immunization:
mock
yes
no
yes
no
Pregnant:
yes
no
Pregnant:
yes
no
Antibody responses to vaccination in pregnant and non-pregnant females; ELISA titers against homologous H1 A/California/07/09 in pregnant females and non-pregnant controls. Serum samples were taken 3 weeks after delivery and samples of females which did not get pregnant were pooled in the experiment where mice were immunized twice. *p<0.05
1 immunization mothers:
Homologous H1 recHA titer do not correlate to litter size

## Slide 2
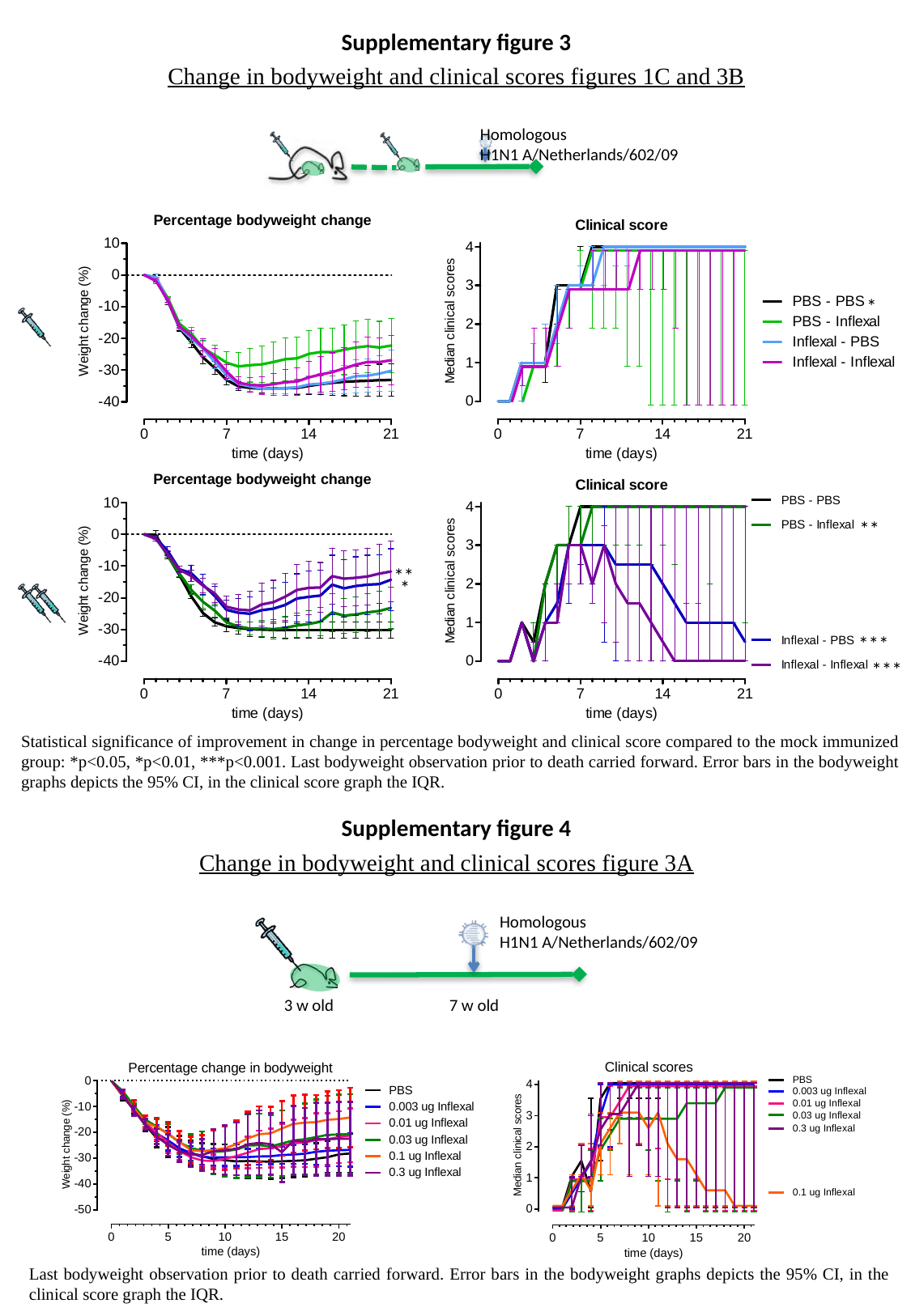

Supplementary figure 3
Change in bodyweight and clinical scores figures 1C and 3B
Homologous
H1N1 A/Netherlands/602/09
*
**
**
*
***
***
Statistical significance of improvement in change in percentage bodyweight and clinical score compared to the mock immunized group: *p<0.05, *p<0.01, ***p<0.001. Last bodyweight observation prior to death carried forward. Error bars in the bodyweight graphs depicts the 95% CI, in the clinical score graph the IQR.
Supplementary figure 4
Change in bodyweight and clinical scores figure 3A
Homologous
H1N1 A/Netherlands/602/09
3 w old
7 w old
Last bodyweight observation prior to death carried forward. Error bars in the bodyweight graphs depicts the 95% CI, in the clinical score graph the IQR.

## Slide 3
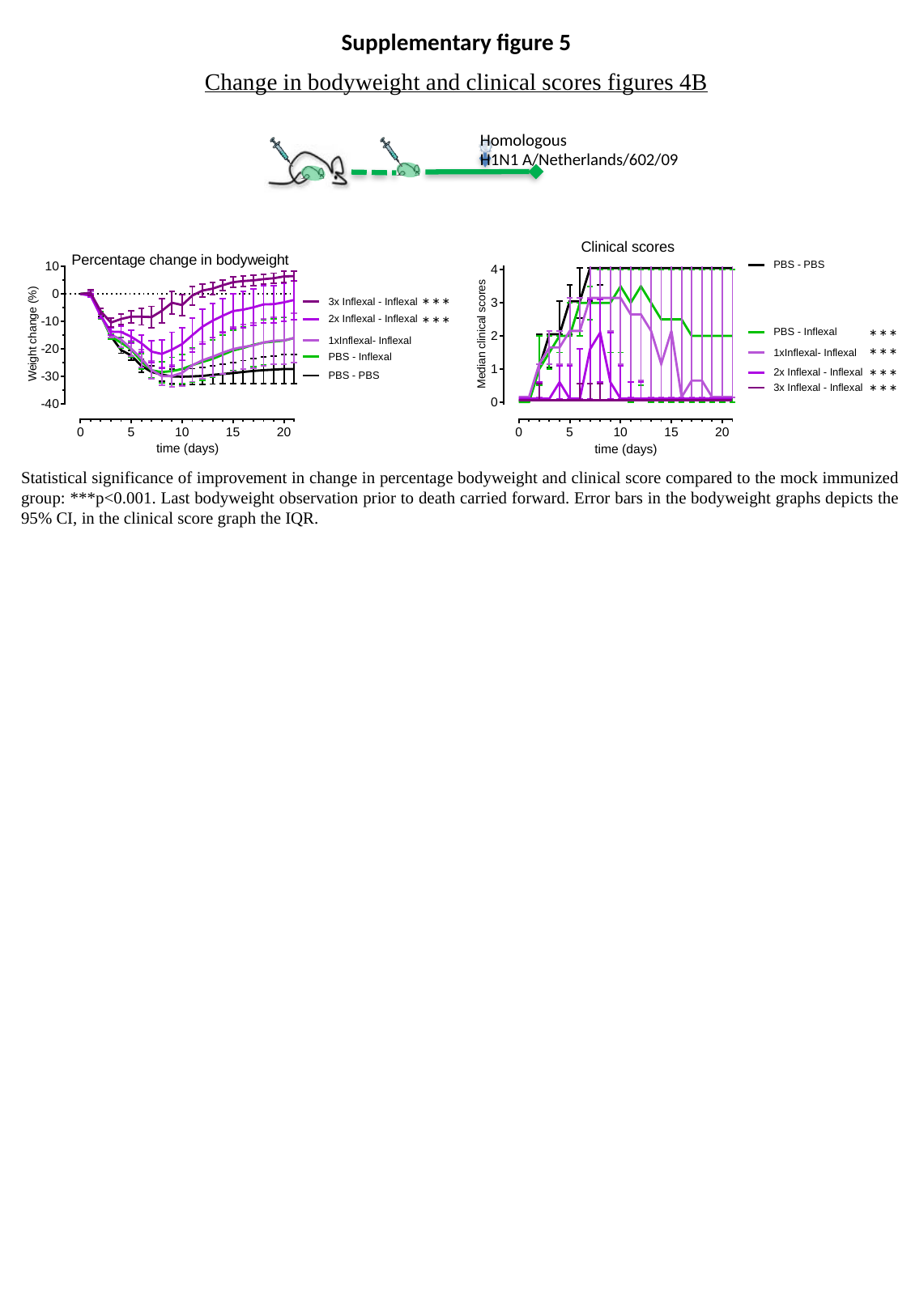

Supplementary figure 5
Change in bodyweight and clinical scores figures 4B
Homologous
H1N1 A/Netherlands/602/09
***
***
***
***
***
***
Statistical significance of improvement in change in percentage bodyweight and clinical score compared to the mock immunized group: ***p<0.001. Last bodyweight observation prior to death carried forward. Error bars in the bodyweight graphs depicts the 95% CI, in the clinical score graph the IQR.
